# Automatic Recognition of Personality Profiles Using EEG Functional Connectivity during Emotional Processing

**DOI:** 10.3390/brainsci10050278

**Published:** 2020-05-03

**Authors:** Manousos A. Klados, Panagiota Konstantinidi, Rosalia Dacosta-Aguayo, Vasiliki-Despoina Kostaridou, Alessandro Vinciarelli, Michalis Zervakis

**Affiliations:** 1 Department of Psychology, University of Sheffield, International Faculty, CITY College, 54453 Thessaloniki, Greece; 2 School of Life and Health Science, Aston University, Birmingham 30511, UK; giotakon.8@gmail.com (P.K.); vcost_92@hotmail.com (V.-D.K.); 3 Department of Electrical and Computer Engineering, Technical University of Crete, 73100 Chania, Greece; michalis@display.tuc.gr; 4 Department of Clinical Psychology and Psychobiology, The University of Barcelona, 731 33 Barcelona, Spain; rdacostaa@gmail.com; 5 School of Computing Science, University of Glasgow, Glasgow G12 8QQ, UK; Vinciarelli@glasgow.ac.uk

**Keywords:** Big-Five factor model, brain functional connectivity, electroencephalogram signal processing, emotional processing, neuroscience, personality detection

## Abstract

Personality is the characteristic set of an individual’s behavioral and emotional patterns that evolve from biological and environmental factors. The recognition of personality profiles is crucial in making human–computer interaction (HCI) applications realistic, more focused, and user friendly. The ability to recognize personality using neuroscientific data underpins the neurobiological basis of personality. This paper aims to automatically recognize personality, combining scalp electroencephalogram (EEG) and machine learning techniques. As the resting state EEG has not so far been proven efficient for predicting personality, we used EEG recordings elicited during emotion processing. This study was based on data from the AMIGOS dataset reflecting the response of 37 healthy participants. Brain networks and graph theoretical parameters were extracted from cleaned EEG signals, while each trait score was dichotomized into low- and high-level using the k-means algorithm. A feature selection algorithm was used afterwards to reduce the feature-set size to the best 10 features to describe each trait separately. Support vector machines (SVM) were finally employed to classify each instance. Our method achieved a classification accuracy of 83.8% for extraversion, 86.5% for agreeableness, 83.8% for conscientiousness, 83.8% for neuroticism, and 73% for openness.

## 1. Introduction

Our work as well as our entertainment, communications, health, security, and education are mainly driven by the advancements made in technology [1]. The way that each user interacts with the computer is affected by his/her personality, which is defined as a relatively stable disposition of an individual that influences his/her behavior [2]. Within this context, researchers have focused their attention on the prediction of personality traits using data collected from online social media, such as Twitter or Facebook [3,4]. Within this scenario, the creation of engaging interfaces, despite the individual differences of the users, has become a challenging goal in the field of human computing interaction (HCI) [1], giving rise to a new field called personality computing [5]. In addition to the aforementioned applications, the recognition of personality using neuroscientific data underpins the neurobiological basis of personality. This modern field of research, which is focused on the relationship between personality traits and cerebral activity [6], is called personality neuroscience [7,8]. A vast amount of behavioral and biological research on personality has raised several theories defining the psychological functions associated with each one of main five personality dimensions [7,9].

The assessment of personality traits based on electroencephalographic (EEG) data in resting-state is far from conclusive. Some studies have reported that resting state EEG can successfully assess personality traits [10], while others have concluded that resting-state EEG spectral power cannot be used to understand the neurobiological substrates of personality [11]. In contrast, other studies have reported that the spectral power of brain oscillations in different frequency bands may serve as a measure of personality [12,13,14,15,16,17]. Most of the research done with EEG has been focused on the relationships between personality traits and EEG alpha activity [14]. However, other studies also support the role of low EEG frequencies (delta and theta activity) in personality traits [15].

Although all these studies have attempted to link personality with resting state EEG using statistical inference, none of them managed to go one step further to use their findings in order to classify or predict different personality traits. There is just one study, conducted by K. Korjus et al. [11], where the authors tried to detect personality from the spectral content of a large dataset of resting-state EEG recordings using a combination of classifiers and features, without achieving a significant classification rate. The authors concluded that the power spectrum of the EEG data could not contribute to the detection of personality traits. 

The spectral analysis of EEG has shed some light on the neurobiological basis of personality, but the most promising results have been obtained when functional connectivity of resting state EEG was taken into consideration. Resting-state connectivity EEG studies have been conducted within the framework of two concepts concerning the dynamic nature of brain networks. The first concept considers that brain networks are static across time [18,19,20,21,22]. As an example, in the study conducted by Toschi et al. [18], the authors reported that conscientiousness (C) was linked to graph theoretical nodal properties of the regions included in the fronto-parietal and the default mode networks. In that study, however, the authors did not find any relation between the functional connectivity and the other four personality traits. The second concept, which is an improvement of the first one, considers that brain networks are dynamic, and time-evolving [23,24]. In the study conducted by Kabbara et al. [23], for example, the authors used a sliding window approach for every subject to analyze the EEG bands in association with personality traits. They found that agreeableness (A) was positively related with the overall centrality variation in the alpha band of the posterior cingulate cortex, while neuroticism (N) was negatively associated with the theta band and with the dynamic variability of temporal lobe regions (left middle temporal gyrus, left superior temporal gyrus, and transverse temporal region). Finally, there was a negative correlation between C and changes in the alpha band. 

The primary purpose of personality neuroscience goes beyond the understanding of the neurobiological systems of traits and focuses on the parameters derived from proximal and distal sources that differentiate one person from another towards constructing personality trait models [25]. Proximal sources refer to neural systems related to the emotional states revealing specific traits, while distal sources describe genetic and environmental factors. Because proximal sources refer to neural systems that are strongly related to emotional states, our main motivation is driven by the assumption that the EEG response elicited during emotional processing may provide features able to predict personality more accurately, reflecting the connection between personality and emotional processing. 

Several studies have appeared in this direction recently. For example, J. Wache et al. [26] conducted an experiment based on physiological responses to automatically detect personality using the Big-Five model. Emotional clips were presented to participants who rated them in terms of valence and arousal. The results supported that baseline accuracy for C and openness (O) was 53%, while for the rest of the traits, it dropped to 50%. The highest recognition accuracy was achieved for O, which varied from 63% up 91%, according to the affective content of the stimuli. In addition, A seems to be a strong predictor for the high valence-high arousal (HVHA) group, scoring 84%. Low recognition performance was observed in C and N with overall accuracy of 31% and 63%, respectively. One limitation of this study, resulting in low accuracy levels, is the use of only one EEG electrode. In another study conducted by Zhao et al. [27], the authors analyzed EEG brain waves with the aim to recognize individual’s personality traits in a sample of 37 participants while they were watching emotional materials. They extracted features from EEG signals and from subjective ratings, which were fed to a support vector machine classifier (SVM) in order to predict the five personality traits’ dimensions. Their model achieved 66.4% accuracy in the classification of extraversion (E), 73.5% in the classification of A, 74.2% for C, 70.4% in the classification for N, and 68% in the classification of O, while the introduction of features from subjective ratings increased the classification accuracy of the model.

The basis of our study builds on (i) our main assumption that personality can be automatically predicted by EEG signals derived during emotional processing, (ii) the study by Zhao et al. [27], and (iii) the fact that functional connectivity studies in resting-state EEG are more promising compared with spectral analysis. Following this motivation, our main goal is to assess if brain networks can be used to automatically predict personality more accurately. For this purpose, the AMIGOS dataset [28] was used herein. Features derived by functional connectivity networks were adopted for classification purposes, because (i) our prior studies [29,30,31,32,33,34,35] suggest that there is an alternation in functional connectivity networks during affective processing, while (ii) to the best of our knowledge, there is no other study that has assessed the value of functional connectivity features in the automatic recognition of personality. ReliefF [36] methodology was adopted in order to rank the produced features, while the first ten common features with the highest ranking were fed to an SVM classifier in order to predict each trait separately. The results herein reinforce our motivation, exhibiting pretty high detection accuracy for all basic five traits (E: 83.8%, A: 86.5%, C: 83.8%, N: 83.8%, and O: 73%). The dataset used in our study, as well as the feature extraction and classification models used, are presented in Section 2. The analytic results are illustrated in Section 3. Critical discussion and conclusions are given in Section 4.

## 2. Materials and Methods

### 2.1. AMIGOS Dataset

**Figure 1:**
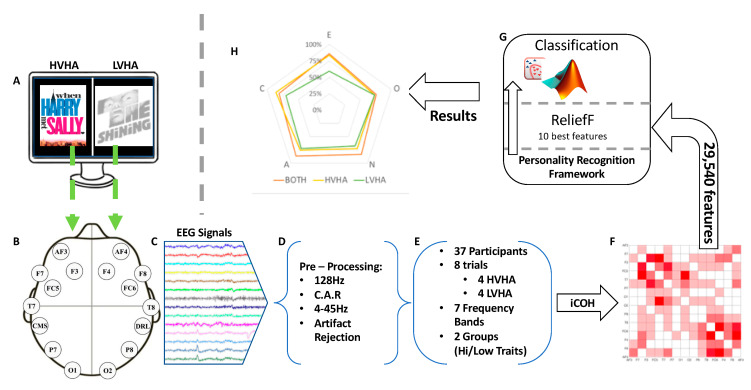
This figure describes the stages of our research methodology. In the beginning, high valence high arousal (HVHA) and low valence high arousal (LVHA) short videos were displayed to the participants of the AMIGOS experiment (**A**) and their affective responses were recorded using the EEG modality (**B**). Then, the EEG recordings (**C**) were pre-processed (**D**), while (**E**) shows the experimental details. The construction of brain networks using iCOH (**F**) followed next and the extraction of features based on the network’s weights as well as on graph theoretical properties was performed. ReliefF (**G**) concluded on the 10 best features per trait, and was used three times; the first time, for sorting the features extracted during HVHA block of clips; the second time, for sorting the features extracted during LVHA block of clips; and one more time, for sorting all the aforementioned features together (BOTH). Finally, the classification stage (**H**) yields the presented prediction accuracy for each one of the five dimensions of personality (neuroticism (N): 83.8%, extraversion (E): 83.8%, openness (O): 73%, agreeableness (A): 86.5%, and conscientiousness (C): 83.8%). EEG, electroencephalographic; iCOH, imaginary part of coherence.

The AMIGOS dataset attempts to approach personality and emotions with a very broad range of features, as it contains information coming from multichannel EEG, electrocardiogram (ECG), and galvanic skin response (GSR) during various affective states, personality questionnaires, self-assessment of mood, and so on [28]. The AMIGOS dataset will be briefly described, as the readers are advised to refer to [28] for more details, while a brief overview of our methodology is presented in Figure 1.

AMIGOS includes two experiments. In the short videos experiment used in the current study, 40 (37 used in the current study owing to missing data) participants watched 16 short videos (duration < 250 s) with emotional content extracted from movies so that specific affective states were elicited. The participants had to self-assess their emotional reaction evoked by a certain video by selecting among the six basic emotions (happiness, sadness, fear, disgust, anger, and neutral), as well as to rate each video by means of valence and arousal. Valence measures positive or negative affectivity, while arousal describes how calm or excited someone is after being exposed to specific stimuli [37]. Considering the two poles of valence and arousal, we formed four categories (high valence high arousal—HVHA, high valence low arousal—HVLA, low valence high arousal—LVHA, and low valence low arousal—LVLA). In our study, we used eight trials of HVHA and LVHA (four trials each). Apart from the experimental process mentioned above, participants’ personality profiles were modeled through the Big-Five inventory [38,39], provided in the form of a self-report online questionnaire with 50 items in a seven-point Likert scale. These 50 questions were divided into five sets, and each set was used to describe one of the five dimensions of personality: N, E, O, A, and C.

Moreover, EEG signals were recorded with an Emotiv EPOC Neuroheadset by 14 channels (AF3, F7, F3, FC5, T7, P7, O1, O2, P8, T8, FC6, F4, F8, and AF4) placed according to the 10–20 system [40]. For the purposes of the current analysis, we used the already pre-processed signals offered by the authors of the AMIGOS database. The sampling frequency was 128 Hz and the signals were high-pass filtered at 2 Hz, while ocular artifacts were removed using blind source analysis [41]. We further applied a new high-pass filter at 4 Hz, so as to remove the delta oscillations from the signals. Delta band was removed, because it is seriously affected by artifacts, more importantly by ocular artifacts. These artifacts cannot be properly rejected, as the AMIGOS dataset has low spatial resolution and lacks electrooculographic signals [42,43]. More details about the pre-processing pipeline are available at http://www.eecs.qmul.ac.uk/mmv/datasets/amigos/readme.html.

### 2.2. Grouping Categorical Variables

**Figure 2:**
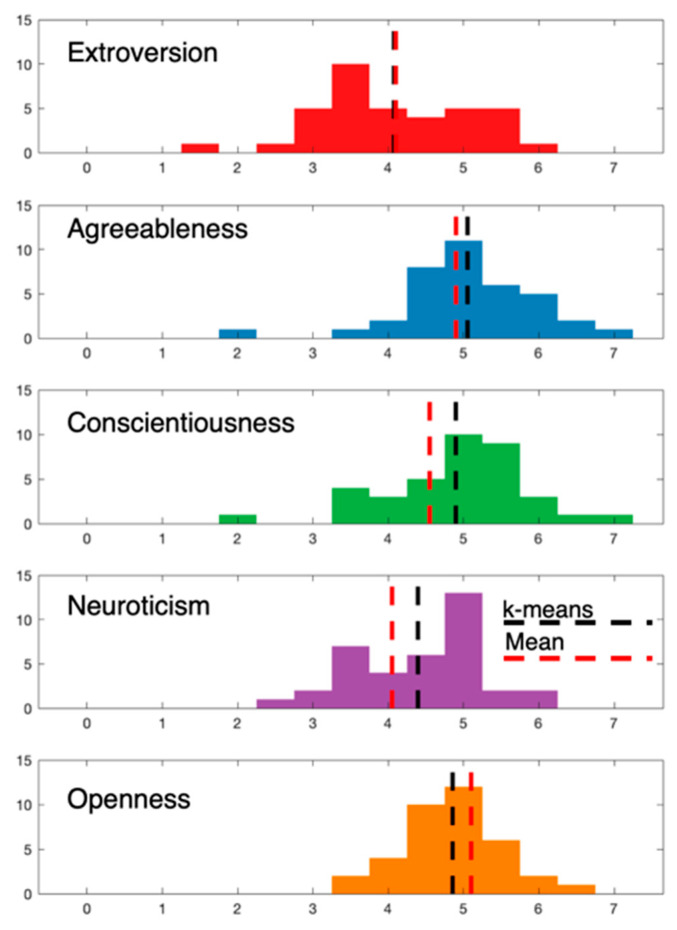
Distributions of the mean scores for each trait. The vertical lines denote the threshold of defining if a score is low or high according to the mean (red) or k-means (black) value of the distribution. In this paper, we used the k-means threshold because it produces groups (low/high) with low differences in respect to size.

In order to reduce the complexity of classifying the personality profiles, we binarized each one of the five personality dimensions into low and high trait. Because the data on every dimension did not follow a normal distribution, the usage of a single threshold (as the mean value of each trait’s distribution) led to remarkable differences between the numbers of instances assigned to each class. Here, we should note that, although the median value can produce classes with an equal number of instances, it is not preferred for replicability purposes [44], as well as for reducing Type-I and Type-II error [45]. Furthermore, the selection of thresholds in the feature space becomes difficult owing to such abnormal trait distribution. For this reason, we exploited the capabilities of unsupervised data-driven clustering and applied k-means for every dimension separately, in order to split our sample into two classes with comparable numbers of instances in the two groups (low/high). The proposed data-driven clustering scheme provides more balanced categories, even though the threshold values in this case differ slightly from those of the mean thresholds (Figure 2 & Table 1).

### 2.3. Functional Connectivity and Graph Modeling

The brain networks were formed from the 14 signals obtained by the EPOC headset, by calculating the imaginary part of coherence [46] for every pair of electrodes. Assuming that $X_{i}$ and $X_{j}$ are two signals with equal sample points, the cross-spectrum is defined as follows: $S_{ij}\left(f\right)=\langle X_{i}\left(f\right)X_{j}^{*}\left(f\right)\rangle$, where * denotes the complex conjugation and 〈 〉 is the expectation value over a sufficiently large number of epochs. Coherence was then defined as the cross spectrum normalized by the spectra of the two aforementioned signals given the following formula:

$$C_{ij}=\frac{S_{ij}\left(f\right)}{\sqrt{S_{ii}\left(f\right)S_{jj}\left(f\right)}}$$

As cross-spectrum is a complex number, coherence is also a complex number. We chose to take only the imaginary part of coherence (iCOH), because Nolte et al. [46] have shown that the imaginary part of coherence is not vulnerable to volume conduction distortions. For the computation of iCOH, we used the FCLAB [47]. As iCOH is computed for every frequency, we averaged the frequencies belonging to a certain brain rhythm (theta: 4–7 Hz, alpha1: 8–9 Hz, alpha2: 10–11 Hz, SMR: 12–14 Hz, beta: 15–29 Hz, and gamma: 30–45 Hz). Thus, for every subject, we computed seven networks—one for each brainwave plus one for the full spectrum (4–45 Hz).

The result of iCOH was a weighted and directed to a network of size 14 × 14. Because the interpretation of directionality in particular frequencies is difficult [46], we transformed the directed networks to a directed one by taking the absolute values of the iCOH matrix. As our new matrix was symmetric, the upper and lower triangular matrices were the same. Thus, we used only the upper triangular matrix, which counts (14 × 14 − 14)/2 = 91 elements that represent the weights of the edges of the network. These 91 elements served as separate features for personality recognition.

From a mathematical perspective, a weighted graph is a mathematical representation of a set of elements (vertices) that may be linked through connections of variable weights (edges). The graph theoretical parameters are quantitative properties of the network that can differentiate one network from another. In addition to the aforementioned 91 features, we added the most prominent graph theoretical parameters, as they can reveal information that is not detectable by single edge weights. A total of 29,540 graph theoretical features were computed using the brain connectivity toolbox [48]. Features are either univariate features indicating the local or global properties of the graph, or bivariate connection features exploring the strength of nodes’ associations. Nevertheless, after the feature selection procedure (see Section 2.4), only the edges’ weights and betweenness centrality (BC) proved to be enough to describe properly the five dimensions of personality. For this reason, we only describe herein the BC and its importance to networks’ efficiency.

BC is the fraction of all the shortest paths in the network that contain a specific node. This parameter is based on the concept that central nodes appear in many short paths and “control” the information flow [49]. The undirected variant of BC of node i is calculated as

$$b_{i}=\frac{1}{\left(n-1\right)\left(n-2\right)}\sum_{\begin{array}{c} h,j\in N\\ h\neq j,h\neq i,i\neq j \end{array}}\frac{\rho_{hj}\left(i\right)}{\rho_{hj}},$$

where $\rho_{hj}$ is the number of shortest paths between h and j and $\rho_{hj}\left(i\right)$ is the number of shortest paths between h and j that include node i [49]. The weighted variant of BC requires the calculation of weighted path lengths.

All the aforementioned features were computed for each one of the eight trials mentioned in 2.1 and then averaged by group (HVHA, LVHA).

### 2.4. Feature Selection

In order to reduce the number of the features, ReliefF algorithm [36] was used. ReliefF was chosen because it is an efficient algorithm that is not restricted by the different characteristics of the dataset, while it can work with both discrete and continuous features. Initially, all features’ weights were set to 0 and a sample $x_{r}$ from the training set was randomly selected. Then, ReliefF finds the k nearest neighbors from each class (in our study k = 10) and for each nearest neighbor $x_{q}$. All the weights for the features $F_{j}$ are adjusted according to the following formula:

$$W_{j}^{i}=\{\begin{array}{c} W_{j}^{i-1}-\frac{\Delta_{j}\left(x_{r},x_{q}\right)}{m}\cdot d_{rq},\;x_{r}\equiv x_{q\;}\\ W_{j}^{i-1}+\frac{p_{y_{q}}}{1-p_{y_{r}}}\cdot \frac{\Delta_{j}\left(x_{r},x_{q}\right)}{m}\cdot d_{rq},\;x_{r}≢x_{q\;}\; \end{array}$$

where $W_{j}^{i}$ is the weight of the feature $F_{j}$ at the $i^{th}$ iteration; $p_{y_{q}}$ and $p_{y_{r}}$ are the prior probabilities of the classes where $x_{q}$ and $x_{r}$, respectively, belong; m is the number of iterations; and $\Delta_{j}\left(x_{r},x_{q}\right)$ is the difference in the values of feature $F_{j}$ between the observations $x_{r}$ and $x_{q}.$ Notice that, in the case of discrete variables (as the personality scores), this difference is given by the following:

$$\Delta_{j}\left(x_{r},x_{q}\right)=\{\begin{array}{c} 0,\;x_{rj}=x_{qj}\\ 1,\;x_{rj}\neq x_{qj} \end{array}$$

where $x_{rj}$ and $x_{qj}$ are the values of the $j^{th}$ feature for the observations $x_{r}$ and $x_{q},$ respectively. Moreover, ≡ denotes that $x_{r}$ and $x_{q}$ are in the same class and $d_{rq}$ is the distance function of the following form:

$$d_{rq}=\frac{\tilde{d_{rq}}}{\sum_{l=1}^{k}\tilde{d_{rl}}}$$

while distance is subject to the scaling $\tilde{d_{rq}}=e^{-{\left(rank\left(r,q\right)/sigma\right)}^{2}}$, where rank(r,q) is the position of the $q^{th}$ observation among the nearest neighbors of the $r^{th}$ observation sorted by the distance.

ReliefF was used in a leave one out fashion during feature selection. This means that we separated the 37 instances (subjects) into 37 sets of 36 instances each. Each one of these datasets was imported to the ReliefF algorithm, which sorted the features according to their score. Then, the frequency of each feature in the 37 iterations was computed and the first 10 more frequent features for each trait were selected. In other words, features that appeared most often within the internal cross validation iterations were selected for each trait.

**Figure 3:**
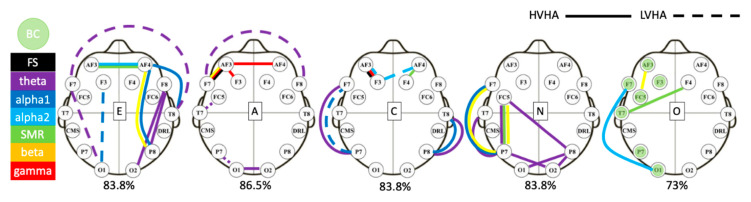
This figure demonstrates the 10 best features extracted from ReliefF algorithm for each dimension of personality as evaluated by the fusion scenario. Bivariate features are demonstrated by lines, whereas univariate features (here only betweenness centrality (BC)) are denoted by colored nodes. The color of each edge represents a specific brainwave (black is full spectrum (FS) from 4 to 45 Hz), while the maximum classification accuracy for each trait is written beneath the head plots. Finally, the solid lines denote features extracted from HVHA clips, while dashed lines denote features extracted by LVHA clips.

The proposed procedure was used three times—one time for sorting the features extracted during HVHA block of clips; a second time for sorting the features extracted during LVHA block of clips; and one more time for sorting all the aforementioned features together (fusion scheme), forming three different feature sets for each one of the five personality’s dimensions. The ten most prominent common features for each dimension and for the fused scenario, allocated per frequency band, are presented in Figure 3. We chose the first ten of the common features, because in almost all cases, they had a significantly higher ranking score than the rest.

### 2.5. Classification

In the current study, the dominant classifier was the SVM, which forms a principled approach to machine learning problems and is considered suitable for binary classification [50], as in our case, where we divided the personality traits to high/low cases.

The mathematical model of linear SVM is defined as follows [51]. We are given a training set ${\left\{y_{i},{\vec{x}}_{i}\right\}}_{i=1}^{l}$, where the input ${\vec{x}}_{i}\;\in {\mathbb{R}}^{n}$ and the output $y_{i}\in \;\left\{-1,+1\right\}$. If there is a hyperplane dividing all samples ${\vec{x}}_{i}$ into groups correctly, the aim is to find the maximum distance between the hyperplane and the nearest point ${\vec{x}}_{i}$ from either group. The optimal hyperplane is then defined by the following classification decision function:

$$f\left(x\right)=sign\left[\overset{l}{\underset{i=1}{\sum }}a_{i}y_{i}\left({\vec{x}}_{i}\cdot \vec{x}\right)+b\right]$$

where $a_{i}\;\geq 0$ are the Lagrangian multipliers of samples ${\vec{x}}_{i}$. Cases with $a_{i}=0$ are not part of the solution. 

When non-linearity is essential to the problem formulation, the only difference compared with the linear model is that we first need to perform data mapping to another high-dimensional space H, using a non-linear mapping called Φ. After that, the linear model is used again to perform classification in the new space H. The kernel function k that is introduced through such a mapping procedure is a symmetric, semi-positive definite function satisfying the Mercer theorem, and converts the previous classification decision function as follows:

$$f\left(x\right)=sign\left[\overset{l}{\underset{i=1}{\sum }}a_{i}y_{i}k\left({\vec{x}}_{i}\cdot \vec{x}\right)+b\right]$$

The Gaussian kernel function as a measure of similarity between $\vec{x}$ and ${\vec{x}}_{i}$ mostly used in our analysis is described as follows:

$$k\left(\vec{x},{\vec{x}}_{i}\right)=\exp\left(-\gamma ||\vec{x}-{\vec{x}}_{i}||{}^{2}\right),\;\mathrm{where}\;\gamma >0$$

It can be observed that the Gaussian kernel depends on the Euclidean distance between $\vec{x}$ and ${\vec{x}}_{i}$ and is based on the assumption that similar points are found close to each other in the feature space [52].

Moreover, two commonly used kernel functions that we also test in our project is the quadratic kernel [52] and the cubic kernel, namely a second and a third degree polynomial kernel function, respectively, are described as follows:

$$k\left(\vec{x},{\vec{x}}_{i}\right)={\left(\vec{x}\cdot {\vec{x}}_{i}+1\right)}^{2}\;\;\mathrm{and}\;k\left(\vec{x},{\vec{x}}_{i}\right)={\left(\vec{x}\cdot {\vec{x}}_{i}+1\right)}^{3}$$

The quadratic kernel not only determines the similarity of input samples, but also examines combination of features up to the order of the polynomial.

Therefore, taking into consideration the benefits of the Gaussian kernel, we chose to apply specific variants of Gaussian SVMs, namely coarse Gaussian SVM, medium Gaussian SVM, quadratic SVM, and cubic SVM. In particular, medium Gaussian SVM performs medium distinctions with kernel scale set to $\sqrt{P}$ and provides medium model flexibility, similarly to quadratic SVM, while coarse Gaussian SVM makes coarse distinctions between classes, with kernel scale set to 4$\sqrt{P}$ and provides low model flexibility. In both cases, P was set to 10, reflecting the number of most significant features.

For validation purposes of the models, fivefold cross validation was used. According to fivefold cross validation, the dataset was randomly partitioned in five equal non-overlapping subsets, where four of them were used for training and one for testing purposes. The cross-validation process was then repeated five times (the folds), with each of the five subsamples used exactly once as the validation data. The five results from the folds were then averaged to produce a single estimation of accuracy.

In addition to accuracy, the prediction of each personality dimension was binarized to low and high. In this form, we considered the successful prediction of a low and high trait as true positive (TP) and true negative (TN), respectively. If the classification failed in the low trait, we considered the sample as a false negative (FN) and, in case of high trait failure, we considered the sample as a false positive (FP). Therefore, accuracy is defined as

$$acc=\frac{\mathrm{TP}+\mathrm{TN}}{\mathrm{Total}\;\mathrm{Population}}$$

while sensitivity is the TP rate given by $\frac{\mathrm{TP}}{\mathrm{TP}+\mathrm{FN}}$ and specificity is the TN rate, $\frac{\mathrm{TN}}{\mathrm{TN}+\mathrm{FP}}$. The area under receiver operating characteristic (ROC) curve (AUC) metric denotes the prediction potential of a classification algorithm for varying classification threshold values and is important in cases of non-homogeneous classes.

## 3. Results

Taking into consideration the suitability and the performance capabilities, this section presents the results mainly derived from SVMs. The assessment of results was based on four main parameters, namely the accuracy, sensitivity, specificity, and AUC that were described above. Table 2, Table 3 and Table 4 present the results regarding the three scenarios, where scenario no. 1 concerns the HVHA block of clips, scenario no. 2 concerns the LVHA block of clips, and scenario no. 3 describes their fusion. The dominant classifier was the medium Gaussian SVM, although in some cases, other classifiers provided slightly higher accuracy.

Table 2 presents the results derived from the features selected by the HVHA block of clips. As can be observed, high valence is a definer of E factor, which scores high prediction accuracy (83.8%).

O and C score lower in accuracy, with O also being described by zero sensitivity, which makes it the weakest predictor for this scenario. In particular, the sensitivity is extremely low for O and C, reflecting the inability of the test to truly detect these conditions. Instead, specificity is low for N and A, indicating low power of the test in ruling out these personality variables.

The LVHA scenario (Table 3) could be described as the weakest test, as it results in the lowest accuracy outcomes for all of the Big-Five dimensions. This was expected, as we are aware that low valence may be more complex for the human brain to perceive and induces the collaboration of multiple brain regions, increasing the process complexity [30]. In this scenario, extroversion also appears with low sensitivity and specificity values.

As we expected, the fusion scheme clearly improves the results regarding all of the examined parameters. Table 4 confirms that the fusion scenario reaches the highest accuracy rates for all of the Big-Five dimensions of personality. The lowest sensitivity parameter concerns again the O trait, though this was expected, as O appears to be the most controversial trait, characterized by high abstractness, unconventionality, thin mental boundaries, and intuition [53].

## 4. Discussion

This study presents innovative research on the detection of personality traits during affective processing by means of neurophysiological signals. The participants’ personality profiles were modeled through the EEG signals obtained by the AMIGOS database. The administration of the Big-Five personality inventory was performed by a self-assessment online questionnaire. The participants were also asked to rate each video according its emotional content in regard to valence and arousal. The most crucial part of the AMIGOS dataset is the use of EEG signals, which are recorded through an easily accessible, wireless, and wearable EEG system (EPOC). With these attributes, this dataset and our proposed analysis framework provide insights into an interdisciplinary aspect by cross-referencing the internal, and hence personal impression of self, with the external neurologically evidenced data derived from emotional reactivity.

From Figure 3, it can be observed that the most important features are the edges in the network except for the O trait, where the nodal BC is dominant. It can also be observed that each personality trait is strongly associated with a characteristic brain region. Starting with A, we observe that it is related with brain activity in the frontal and the occipital lobes with a dominant theta frequency band. As for N, it is mostly detected in the parieto-temporal lobe, probably owing to the hippocampus involvement, with a left-side dominance while increased theta activity is also detected.

Furthermore, as mentioned before, the O trait is strongly associated with BC. More specifically, increased BC implies an enhanced coordination of brain networks because it plays a significant role in information transition and controls the information flow. C covariates with the communication in the lateral prefrontal cortex and both parietal lobes, while E is associated with increased connectivity in the frontal and left temporal lobes mainly detected in alpha and theta frequency bands.

As we have already mentioned in the introduction, Zhao and colleagues [27] have recently attempted to predict personality from EEG signals during emotional processing. Our main differences from their work can be seen in the fact that our study uses a low-cost EEG device while Zhao et al. [27] have used professional equipment, which cannot be applied in real world applications aimed at to the average end-user. On the other hand, considering the complex nature of emotions and emotional processing, we used features derived from multivariate modelling, like brain networks. Our assumption, that the brain networks may serve as a better feature-set for the automatic recognition of personality, is actually supported by our classification results, which, in comparison with Zhao et al. [27], are a bit higher in terms of accuracy (E: 83.8% (Zhao: 66.4%), A: 86.5% (73.5%), C: 83.8% (74.2%), N: 83.8% (70.4%), O: 73% (68%)). We should emphasize here that the obtained results based on brain networks perform better than the spectral features reported in Zhao et al. [27], even though the underlying data suffer in quality as they are obtained from low-cost equipment. This performance further highlights the potential of second-order correlation features in the analysis of EEG signals.

Taking into consideration that N is intertwined with the expression of negative emotions such as feeling worried and anxious, while extroversion is associated with positive emotions as optimism and pleasure from social interactions, our results imply that this susceptibility towards positive or negative states may come as a consequence of personality traits, and vice versa [54]. From a mathematical perspective, this means that the observed variance in brain’s emotional reactivity (like event related potentials (ERPs) [55], event related oscillations (EROs) [56,57], functional connectivity [34], and so on) may be explained by the variance observed in personality traits, and vice versa. For this reason, we may assume that human personality can be more clearly predicted from the EEG response during elicitation of high arousing emotional stimuli, as low arousing stimuli result in small (or insignificant) inter-individual variability [55]. This is the main reason that low arousal categories were excluded for the current work. Furthermore, the relationship between valence and arousal is more complicated than one could expect. For example, anger and joy are similar regarding their emotional arousal, but completely opposite in terms of valence. Besides that, co-variation of arousal with valence is particularly strong in the case of negative images, which tend to be rated as more arousing than positive images [58]. Despite these considerations, dimensional and discrete perspectives differ in how emotional states are conceptualized and described (see [59]). Hence, the inclusion of arousal as an independent variable is expected to create more problems than opportunities in the classification of the five traits of personality.

The personality–affect relationship has generated great attention since it was proposed in Eysenck’s personality model [60]. Eysenck claimed that E, the personality dimension that describes a person as either communicative or uncommunicative, is related to low cortical arousal—that is, extraverts require more external stimulations than introverts [61]. His model also proposed that neurotics were more sensitive to external stimulation and become easily nervous in the presence of minor life stressors.

Many affective studies have attempted to validate and extend Eysenk’s findings [62]. Nevertheless, few works have investigated affective correlates of traits other than E and N owing to the complexity of the personality construct, as many factors should be taken into consideration in order to properly classify an individual according to the initial model described in [60]. This situation complicates the use of affective recognition models in classifying personality traits.

In our work, we address the hypothesis that EEG features during emotional processing can provide strong associations to effectively predict the five personality traits. For this purpose, we used a dataset that involves affect and personality traits [28]. The main findings revealed high detection accuracy for all of the Big-Five personality traits (N: 83.8%, E: 83.8%, O: 73%, A: 86.5%, and C: 83.8%), thus confirming the initial hypothesis that personality features can be automatically predicted by EEG during emotional stimulation. These findings appear to be consistent with the findings of prior studies of brain activation patterns in relation to emotional states that presented a specific display of physiological responses as indicative of equivalent personality features [22]. N is typically assessed with items emphasizing affective content; E, A, and C emphasize behavioral content; and O is represented primarily by cognitive content [63,64]. This could explain our results related to specificity in classifying every dimension/trait, which were lower for O and higher for N, A, E, and C.

Increased attention should be paid in O, when someone comes to interpret our results, as O has very low (0–0.1) sensitivity. This happened because the classification algorithm failed to detect the low case and assigned all samples to the high class. In order to further investigate if this problem is data- or algorithm-specific, we tested several other classification algorithms with very similar results. However, considering the O values (Figure 2), it can be noticed that their distribution is normal (not right skewed as the rest) and shifted to the right end with only small variation. Indeed, all of the subjects used in the current study have high values of O (4.8541 ± 0.6644), although some of them were assigned to a low class because the k-means algorithm forced to separate them into two classes.

In light of these results, it is important to mention certain limitations reflected in the interpretation of our findings in order to gain a holistic understanding of the algorithmic potential. The first issue is that personality is concerned with affect, behavior, cognition, and desire [65]. Further studies focused on the recognition of personality profiles should take into consideration not only the affective, but also the behavioral and cognitive features to improve the performance of classification algorithms. The second one is that affect is described as a higher-order functionality, subsuming valence conditions such as moods, emotion, feeling states, and preferences [64,66]. Further studies with larger sample populations should take into consideration not only the factors mentioned, but also the ability to differentiate emotion from mood in order to improve the classification performance. Moreover, personality is evidenced in terms of thinking, feeling, and behaving, thus self-reflection and introspection are considered to be of paramount importance in self-assessment and self-evaluation. Taking into consideration that the AMIGOS participants coincide with the individuals that complete the self-assessment questionnaire, the results may have been inevitably subjectivity-biased as well as susceptible to self-preservation cognitive attributions.

It has to be mentioned here that the self-assessment questionnaires are generally perceived as weak predictors of traits owing to the biased perception of self, while the answers may be intentionally misleading for various reasons, feeding the HCI system with wrong information about personality. In this manner, EEG is considered as an unbiased and more accurate method of personality recognition, whose operation is difficult to be intentionally altered.

The present findings reflect an added value to the relevant previous studies that examined personality traits through only self-assessment questionnaires. This paper introduces a neuroscientificaly established evaluation of the personality features, which can significantly contribute to the further study of traits and developmental characteristics. This can particularly advance the theories of personality, with a specific focus on (i) how adverse or powerful negative experiences affect our personality development and (ii) more comprehensive understanding of how one’s current disposition may affect the coping strategies with reference to emotion regulation.

Our results open new paths in dealing with emotional states and their association to (or aspects of) personality. Besides the study of issues affecting personality and its consequences on the effectiveness of HCI systems, our proposed methodology may find applications in psychology studies towards best practices for improving life standards depending on an individual’s personality. In general, we may argue that the classification of personality traits should be a priority when applying early cognitive, emotional, and behavioral techniques to improve the attitude and overall quality of life of people who experience problems originating from personality, either related to clinical factors (affective and/or personality disorders) or of a non-clinical nature [67].

## Figures and Tables

**Table 1:** Number of instances.

	k-Means		Mean	
Dimension	Low	High	Low	High
Extroversion	20	17	20	17
Agreeableness	18	19	19	18
Conscientiousness	16	21	17	20
Neuroticism	17	20	12	25
Openness	27	10	26	11

**Table 2:** Results for the high valence high arousal (HVHA) scenario. AUC, area under ROC curve.

Dimension	Accuracy	Sensitivity	Specificity	AUC
Extroversion	83.8%	0.82	0.85	0.86
Openness	64.9%	0	0.89	0.67
Neuroticism	78.4%	0.92	0.54	0.84
Agreeableness	75.7%	0.90	0.56	0.82
Conscientiousness	67.6%	0.15	0.96	0.73

**Table 3:** Results for low valence high arousal (LVHA) scenario.

Dimension	Accuracy	Sensitivity	Specificity	AUC
Extroversion	56.8%	0.47	0.65	0.55
Openness	73%	0	1	0.71
Neuroticism	64.9%	0.96	0.08	0.64
Agreeableness	70.3%	0.81	0.56	0.71
Conscientiousness	62.2%	0.31	0.79	0.72

**Table 4:** Results for fusion scheme.

Dimension	Accuracy	Sensitivity	Specificity	AUC
Extroversion	83.8%	0.82	0.85	0.86
Openness	73%	0.1	0.96	0.74
Neuroticism	83.8%	0.92	0.69	0.89
Agreeableness	86.5%	0.90	0.81	0.92
Conscientiousness	83.8%	0.6	0.88	0.77
